# Protist communities of microbial mats from the extreme environments of five saline Andean lagoons at high altitudes in the Atacama Desert

**DOI:** 10.3389/fmicb.2024.1356977

**Published:** 2024-03-20

**Authors:** Eduardo Acosta, Frank Nitsche, Cristina Dorador, Hartmut Arndt

**Affiliations:** ^1^Department of General Ecology, Institute of Zoology, University of Cologne, Cologne, Germany; ^2^Department of Biotechnology, Universidad de Antofagasta, Antofagasta, Chile; ^3^Centre for Biotechnology and Bioengineering (CeBiB), Universidad de Antofagasta, Antofagasta, Chile

**Keywords:** microbial mats, metabarcoding, protists, co-occurrence, *Rhogostoma*, *Euplotes*, salinity

## Abstract

**Introduction:**

Heterotrophic protists colonizing microbial mats have received little attention over the last few years, despite their importance in microbial food webs. A significant challenge originates from the fact that many protists remain uncultivable and their functions remain poorly understood.

**Methods:**

Metabarcoding studies of protists in microbial mats across high-altitude lagoons of different salinities (4.3–34 practical salinity units) were carried out to provide insights into their vertical stratification at the millimeter scale. DNA and cDNA were analyzed for selected stations.

**Results:**

Sequence variants classified as the amoeboid rhizarian *Rhogostoma* and the ciliate *Euplotes* were found to be common members of the heterotrophic protist communities. They were accompanied by diatoms and kinetoplastids. Correlation analyses point to the salinity of the water column as a main driver influencing the structure of the protist communities at the five studied microbial mats. The active part of the protist communities was detected to be higher at lower salinities (<20 practical salinity units).

**Discussion:**

We found a restricted overlap of the protist community between the different microbial mats indicating the uniqueness of these different aquatic habitats. On the other hand, the dominating genotypes present in metabarcoding were similar and could be isolated and sequenced in comparative studies (*Rhogostoma, Euplotes, Neobodo*). Our results provide a snapshot of the unculturable protist diversity thriving the benthic zone of five athalossohaline lagoons across the Andean plateau.

## Introduction

Microbial communities thriving in hostile environments, mainly composed of so-called extremophiles, represent hotspots of biodiversity and harbor invaluable genetic resources, which are the key to answering fundamental questions about the limits of life (Warren-Rhodes et al., 2006; Schulze-Makuch et al., 2018). Given the vast diversity of such communities, it is difficult to assess how they will respond to disturbances and to predict the possible functional consequences for the ecosystem. A significant challenge originates from the fact that many microbes remain uncultivable and their functions remain poorly understood (Solden et al., 2016). However, modern sequencing techniques allow for the exploration of the genetic pool of microbial communities, the study of their structure and functions, and the examination of biogeographic patterns in response to contemporary environmental factors and historical contingencies (Venter et al., 2017; Schiwitza et al., 2018, 2019, 2021; Arndt et al., 2020; Rybarski et al., 2023).

High-altitude lagoons in the Andean Altiplano (15°-22° S, up to 6.170 m a.s.l.) belong to endorheic basins and include diverse microbial ecosystems such as lagoons, brines, wetlands, and salars composed of plankton and benthos enduring hostile conditions (Albarracín et al., 2015; Aran et al., 2021). Part of these microbial guilds is formed by microbial mats, which colonize precipitating salts and rocks within such ecosystems. They interact with physical-chemical gradients, such as the varying mineral concentrations characteristic of Andean water bodies, distributed throughout the ancient Atacama Desert (Demergasso et al., 2003; Farías et al., 2014; Farias et al., 2017; Rasuk et al., 2014, 2016; Fernandez et al., 2016; Saghaï et al., 2017; Vignale et al., 2022). Microbial mats have been identified as important sites for greenhouse gas exchange and complex variable ecosystems in the athalossohaline Andean salt flats, linking these complex laminar assemblages to the cycling of carbon in the remote Altiplano plateau (Dorador et al., 2018; Molina et al., 2021). The microbiology of these stratified microhabitats has been linked mainly to primary producers (cyanobacteria) and is considered to be among Earth's earliest ecosystems (Margulis et al., 1980; Dupraz et al., 2009). Various Andean microbial mats have been studied, focusing on their prokaryotic communities, including archaea and a wide range of viruses, exhibiting changes along the vertical gradient (e.g., Dorador et al., 2008, 2013; Hernández et al., 2016; Eissler et al., 2019). Considering protists, unicellular eukaryotic biosignatures have mainly been described from lower altitudes (Salar de Llamará, 750 m a.s.l.), in saline microbial mats and hypersaline lagoons harboring biosignatures classified as diatoms, bicosoecids, and ciliates as detected by metabarcoding (Saghaï et al., 2017; Rybarski et al., 2023). Efforts to isolate and sequence the cultivable and uncultivable protist diversity from saline to hypersaline lagoons unveiled a novel endemic diversity including species from Opisthokonta (choanoflagellates), Rhizaria (Cercomonadida), Discoba (percolomonads), Stramenopiles (bicosoecids and placidids), and Alveolata (ciliates) (Schiwitza et al., 2018, 2019, 2021; Arndt et al., 2020; Carduck et al., 2021; Rybarski et al., 2021, 2023; Schoenle et al., 2022; Acosta et al., 2023; Hohlfeld et al., 2023).

The increasing focus on protists all over the planet has unveiled a heterogeneous and variable diversity that can be found across different biotopes (terrestrial to aquatic), unveiling biogeographical patterns even within ecosystems (Dillon et al., 2009; Mahé et al., 2017; Schoenle et al., 2021; Singer et al., 2021; Rybarski et al., 2023). Protists have been found to follow the vertical stratification characteristics in microbial mats, assembling differentially relative to vertical physic-chemical gradients (Saghaï et al., 2017). Under the current climate change scenario, water availability is being threatened, highlighting the importance of studying the structure of protist communities living in delicate ecosystems such as the Andean microbial mats. In this study, we assess the structure of these communities at the vertical millimeter scale in the microbial mats of five Andean Lagoons using metabarcoding and explore their differences and their interaction with the overlying water column.

## Materials and methods

### Sample collection and molecular biology procedure

**Figure 1:**
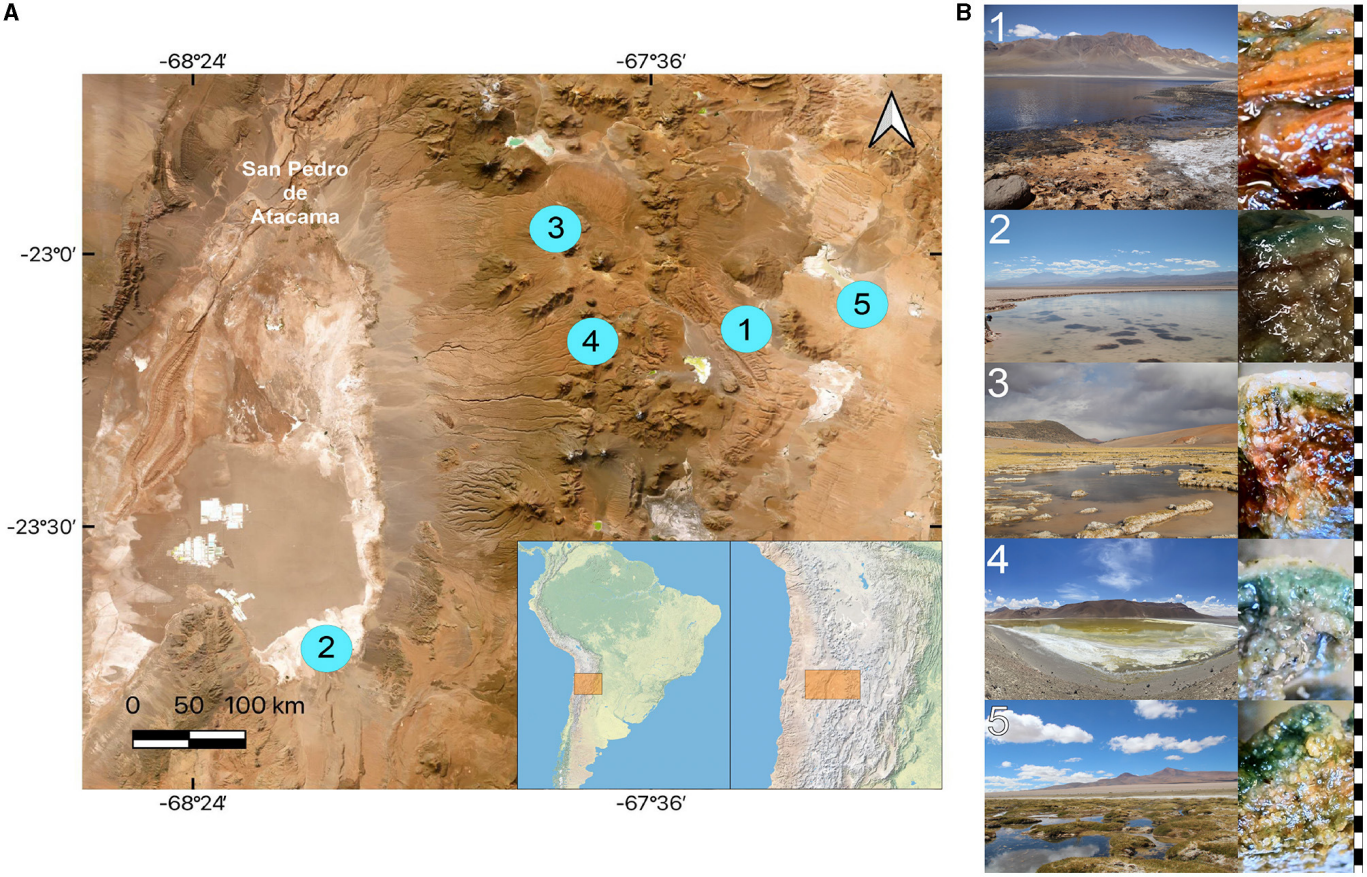
Sampling sites of microbial mat studies in the Atacama Desert. **(A)** Map showing the location of the stations. **(B)** Study sites and vertical structure of the corresponding microbial mats. 1, Aguas Calientes Salar; 2, Salada Lagoon. 3, Quepiaco Salar; 4, Amarilla Lagoon; 5, Helada Lagoon. Right side: scale in black and white indicating one millimeter intervals. Map produced and adapted from QGIS 3.28.5 - Firenze.

Microbial mats were sampled from the sediments below the water line in five lagoons at different altitudes across the Chilean Altiplano (Table 1). The structure of the studied microbial mats differs visibly throughout the studied sites (Figure 1B). Microbial mat layers characterized by an upper green layer were found in Helada Lagoon, Amarilla Lagoon, and Salada Lagoon, while in Quepiaco Salar, microbial mats are covered by a layer of salt and the upper green layer is thinner. In contrast, the microbial mat sampled from Aguas Calientes Salar exhibits a structure of orange and red layers. Biological samples of the microbial mats were dissected *in situ* using sterile metalware and coveralls to maintain cross-contamination to a minimum. The microbial mats were dissected to obtain three biological samples from each layer, starting from the upper layer (first millimeter), the second layer (3 mm depth), and a third layer (5 mm depth; Figure 1). The environmental parameters of the water column, such as salinity (Practical Salinity Units, PSU), resistivity (Ohm/cm), conductivity (mS/cm), and total dissolved solids (parts per trillion), were measured using an Orion Star A322 Conductivity Portable Multiparameter (Thermo Scientific) (Table 1). The sediment and water temperatures were measured using a HI935002 Dual Channel K-Type Thermocouple Thermometer (Hanna Instruments). Each replicate was homogenized in RNA later to preserve the DNA and RNA from each sample (Wang et al., 2018) in sterile 2-ml Eppendorf tubes and stored at −20°C until further analyses. The total DNA and RNA were extracted from samples using Trizol (Invitrogen), according to the manufacturer's protocol with minor modifications: Samples were homogenized in 1 ml of Trizol reagent for 10 min as a cell lysis step using a vortex adaptor. The final DNA was resuspended in 100 μl of nuclease-free water and used immediately for the amplification of the target gene. RNA was resuspended in 50 μl of nuclease-free water for downstream analyses. Total RNA was used as a template for cDNA synthesis using the RevertAid First Strand cDNA Synthesis Kit (Thermo Fisher). Total concentrations of DNA, RNA, and cDNA were measured using the ND-1000 nanodrop (Peqlab Biotechnologie, Germany).

**Table 1:** Information of the stations indicating geographical and physic-chemical properties at each station.

**Station**	**Altitude (m a.s.l.)**	**Geographic location (latitude/longitude)**	**Water temperature (°C)**	**Sediment temperature (°C)**	**Practical salinity units (PSU)**	**Resistivity (Ohm/cm)**	**Conductivity (mS/cm)**	**Total dissolved solids (TDS)**
Helada Lagoon	4.306	23°0605.3"S/67°0837.1"W	25.1	24.36	4.51	124.94	8.06	3.9
Amarilla Lagoon	4.537	23°1312.432S/67°3615.39W	15.5	17.08	15.51	146.01	23.21	11.37
Salada Lagoon	2.297	23°4105.2"S/68°0821.0"W	27.2	25.75	34	19.45	51.42	25.1
Aguas Calientes Salar	4.232	23°0848.2"S/67°2509.1"W	50.4	46.11	24.92	25.71	38.89	19.06
Quepiaco Salar	4.580	23°0456.6"S/67°3555.4"W	10.1	11.5	7.95	69.93	14.3	7.07

### Mock community preparation and sequencing

Aiming to assess the accuracy of the used classification method for sequencing libraries, we included an artificial sample (a mock community). This sample was composed of equimolar concentrations of the PCR-amplified V9 region from eight species from the Heteroflagellate Collection Cologne (HFCC) (Schoenle et al., 2021). The control samples included the V9 region (rDNA) amplicon aliquots of strains from the six protist supergroups Alveolata, Ancyromonadida, Rhizaria, Discoba, Stramenopiles, and Opisthokonta: *Protocruzia* sp. (MT355146), *Aristerostoma* sp. (MT081566), *Fabomonas tropica* (MT355148), *Massisteria* sp. (MT355122), Rhynchomonadidae sp. (MT355133), *Cafeteria burkhardae* (MN315604), Bicosoecida sp. (MT355117), and *Ministeria vibrans* (MT355150) (Supplementary Table 1). For this, the DNA was extracted from the strains by transferring clonal cultures (30 ml) into 50-ml centrifuge tubes (Sarstedt, Nümbrecht, Germany) and centrifuging at 4,000 × *g* at 4°C for 20 min. After discarding the supernatant, the DNA was extracted from the cellular pellet using the Quick gDNA Mini-Prep Kit (Zymo Research Corporation, United States) following the manufacturer's instructions. An aliquot of 3 μl of DNA from each strain was used as a template in a PCR reaction using 5 μM of the universal primer set 18S: Forward (5-AACCTGGTTGATCCTGCCAGT-3) and Reverse (5-TGATCCTTCGCAGGTTCACCTAC-3) (Medlin et al., 1988). The PCR conditions were the following: a denaturation step at 96°C for 120 s, then 34 cycles of 96°C for 30 s, 55°C for 45 s, 72°C for 150 s, and a final elongation step for 420 s at 72°C. The PCR products were purified using the FastGene Gel/PCR Extraction Kit (Fast Gene, Japan), and the sanger was sequenced using the 18S rDNA primer sets at GATC Biotech, Germany. The assembled sequences of the 18S rDNA gene were analyzed using the Basic Local Alignment Search Tool (RRID:SCR_004870).

### Amplification of the hypervariable region V9 SSU rDNA and next-generation sequencing

The aliquots of the DNA and cDNA from environmental samples as well as from the DNA isolated from the strains for the mock community were used as a template for the amplification of the hypervariable region V9 of the small sub-unit 18S rDNA. The amplification was carried out using the multiplex identifier-tagged (MIDs) primers 1389F and 1510R, following the PCR protocol of Amaral-Zettler et al. (2009). The PCR conditions were the following: 98°C for 30 s; 25 cycles of 10 s at 98°C, 30 s at 57°C, 30 s at 72°C, and a final step at 72°C for 600 s. The PCR products were purified using the FastGene Gel/PCR Extraction Kit (Fast Gene, Japan), and equimolar concentrations of all purified PCR products were pair-end sequenced (2 × 150 bp) in multiplexed libraries using an Illumina NovaSeq 6000 sequencer (Illumina, United States) at the Cologne Center for Genomics (CCG).

### Bioinformatic analyses

Multiplexed libraries of the reads obtained through the metabarcoding of DNA and cDNA were processed in QIIME2 (RRID:SCR_021258, Bolyen et al., 2019). The MIDs and primer sequences were removed from the libraries using the “cutadapt trim-paired” function, and sequences with an error rate higher than 0 were filtered. The raw forward and reverse reads were trimmed to exclude the primer sequences, dereplicated, and denoised into amplicon sequence variants (ASVs), using the “denoise-paired” function from the DADA2 plugin (RRID:SCR_023519, Callahan et al., 2016). This method includes a chimera-filtering step on the ASVs using the “consensus” chimera detection method on all samples, and sequences found chimeric in a sufficient fraction of samples were removed (method: isBimeraDenovo). To discern between protist taxa, the ASVs were classified using a cut-off value of 0.8 and a 97% identity to the database through the function “feature-classifier classify-consensus-blast” (Camacho et al., 2009). For this, we used the Protist Ribosomal Reference (PR^2^) database version 4.14.0, amended with the V9 region of the 18 SSU rRNA gene sequences from the Heterotrophic Flagellate Collection Cologne (HFCC) (Schoenle et al., 2021). The ASVs classified as metazoa, archaeplastida, fungi, or unassigned were excluded from further analyses.

### Statistics and reproducibility

The alpha and beta diversity metrics were obtained through the function “qiime diversity core-metrics-phylogenetic”, which relates to the lowest number of sequences. The difference in the alpha-diversity metrics (observed number of ASVs, the Shannon index) across the study sites was tested in a Kruskal-Wallis test for paired groups (study site-study site and DNA-cDNA) using the function “qiime diversity alpha-group-significance”. Additionally, we tested the dissimilarity in beta diversity metrics (Unifrac metrics, Jaccard, and Bray-Curtis distances) across study sites in a permutational analysis of variance (PERMANOVA, 999 permutations) through the function “qiime diversity beta-group-significance”. The output files, such as the frequency table, the sequences table, the phylogenetic tree, the assigned taxonomy, and the metadata used in QIIME2, were imported into R (version 4.3.0) and processed using the “microeco” package version 1.1.0 (Liu et al., 2021). The bar plots showing the relative abundance of the major protist lineages across study sites, as well as clustering patterns based on Euclidean distances, were plotted using the “plot_bar”, setting the clustering option “TRUE”. The beta diversity patterns observed through Jaccard distance were analyzed in a principal coordinate analysis (PCoA) for the DNA and DNA-cDNA-based protists community (97% of identity to the database), using the functions “cal_ordination” and “plot_ordination”. Venn diagrams were generated to depict the number of shared and unique ASVs across microbial mat layers. Furthermore, a canonical redundancy analysis (RDA) with feature selection was used to evaluate the association between the environmental parameters measured in the water bodies (independent variables) and the protist taxa (dependent variables), detected through DNA and cDNA, respectively. Due to the presence of null values, the data was normalized, scaling the abundance of the ASVs to unit variance and adding “scale = TRUE” in the “cal_ordination” function.

## Results

Overall, all the studied water bodies are mesohaline, ranking from 4.51 PSU (Helada Lagoon) up to 34 PSU (Salada Lagoon, Atacama Salar). Other parameters measured in the water column are shown in Table 1.

### Next-generation sequencing analysis

It was planned to have DNA and cDNA data sets from all sampling sites. However, cDNA could not be amplified from all stations. We successfully amplified the target phylogenetic marker mainly from the upper layers of a total of 23 microbial mat samples, distributed as follows: four samples from Helada Lagoon, three from the first layer and one from the second layer; a total of seven samples from Quepiaco Salar, three from the first layer, three from the second layer, and one from the third layer; a total of five samples from Amarilla Lagoon, three samples from the first layer and two samples from the second layer; from Aguas Calientes Salar, a total three samples from the first layer; and from Salada Lagoon, three samples from the first layer. We amplified the target gene from three out of five study sites in a total of 15 samples for cDNA. From Helada Lagoon, there were a total of four samples: one from the first layer, two from the second layer, and one from the third layer. From Quepiaco Salar, there were a total of seven samples: three from the first layer, three from the second layer, and one from the third layer. From Amarilla Lagoon, there were a total of four samples: two from the first layer and two from the second layer.

A total of over 54.6 million raw forward and reverse reads were obtained from the DNA metabarcoding of the five studied microbial mats, of which over 53.1 million (97.2%) were barcode-clipped. After quality control and denoising, a total of over 41.8 million sequences associated with 32,089 ASVs were obtained. For cDNA, a total of 24.7 million raw forward and reverse reads were obtained from three out of five of the studied microbial mats (Helada Lagoon, Amarilla Lagoon, and Quepiaco Salar). From these, ~17.5 million (70.82%) were barcode-clipped, and after quality control and denoising, we obtained a total of ~13.9 million sequences associated with 19,134 ASVs. As we included an artificial control sample of known composition (the so-called mock community), we could assess the accuracy of the taxonomic classification method used in this work. The sequences for each of the eight taxa of the mock community that could be assigned to the respective taxon contributed 3.1–24.4% to the total abundance of reads, using a diversity metric commonly used in environmental sequencing assessments (97% of identity to the database). Additionally, we detected low-frequency ASVs classified as taxa that were not included in the mock community. Such taxa were found at frequencies ranging from 0.032 to 0.047% in the different sequencing libraries. These percentages were used as a threshold for each library preparation, aiming to exclude potential spurious sequences of low abundance.

After applying the threshold from the mock community, our environmental samples include a total of over 37.7 million sequences (90.3% of the total sequences) associated with 4,868 ASVs (15.17% of the total ASVs), remaining from the DNA metabarcoding (Table 2), and a total of over 13.2 million sequences (94.8% of the total sequences) associated with 4,835 ASVs (25.2% of the total ASVs) remained from the cDNA metabarcoding. Using a 97% identity to the database, we identified 308 ASVs associated with 42 lineages (6.3% of the remaining ASVs after filtering) from the DNA metabarcoding, and these were associated with over 14.2 million sequences (17.6% of the remaining sequences after filtering). From the cDNA metabarcoding, we identified a total of 386 ASVs associated with 41 lineages (7.9% of the remaining ASVs after filtering), which were associated with 747,027 sequences (5.6% of the remaining sequences after filtering).

**Table 2:** The amounts of sequences and ASVs of DNA and cDNA origin obtained at critical steps of the bioinformatic analysis used in this study.

**Template**	**Station**	**Samples**	**Total reads**	**Total ASVs**	**Reads after filter**	**ASVs after filter**	**Protist reads**	**Protist ASVs**	**Protist reads (after filter)**	**Protist ASVs (after filter)**
DNA	Helada Lagoon	4	4,623,819	7,565	4,115,878	972	1,080,626	380	1,050,871	69
	Amarilla Lagoon	4	7,686,612	4,833	6,942,574	796	1,966,195	287	1,938,534	107
	Salada Lagoon	4	8,998,521	3,335	7,343,385	353	4,673,791	301	4,664,371	61
	Aguas Calientes Salar	4	6,344,391	3,926	6,050,773	379	3,639,749	222	3,621,420	42
	Quepiaco Salar	7	14,164,719	12,430	13,320,665	2,368	93,460	447	65,742	29
cDNA	Helada Lagoon	4	6,564,350	7,753	6,210,626	1,178	601,112	392	181,887	181
	Amarilla Lagoon	4	2,563,837	3,111	2,486,032	1,223	123,313	245	118,339	115
	Quepiaco Salar	6	4,848,733	8,270	4,566,137	2,434	48,239	320	40,628	90

The protist reads and ASVs correspond to the pre-filter assessment.

### Protist community structure in Andean microbial mats

**Figure 2:**
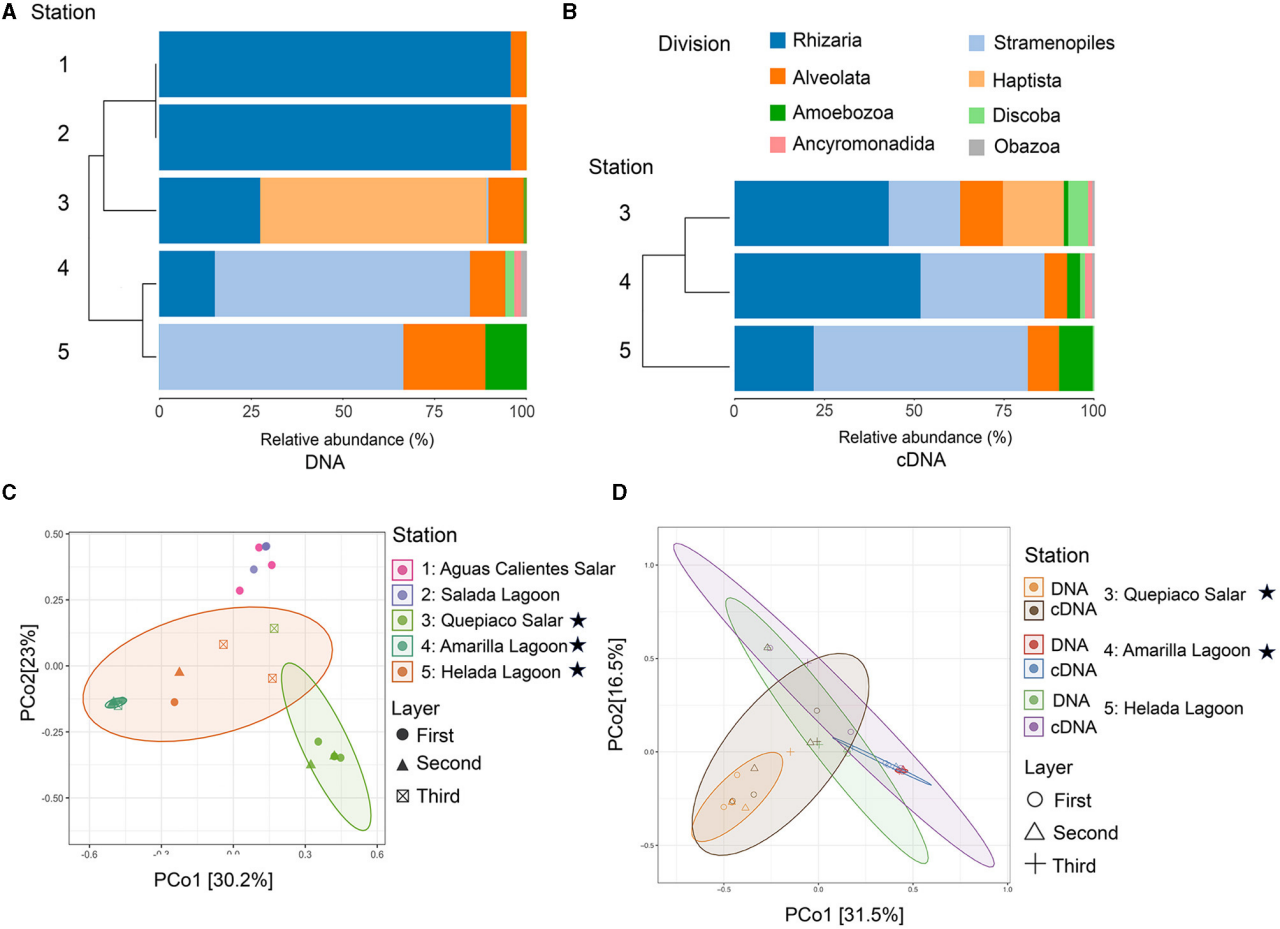
Diversity of protist taxa in microbial mats based on NGS data. **(A)** Cluster analysis and relative abundance of the major protist lineages detected through DNA (identity to the database > 97%) across the five stations. **(B)** Cluster analysis and relative abundance of the major protist lineages detected through cDNA (identity to the database > 97%) across the study sites 3, 4, and 5. Stations, 1, Aguas Calientes Salar; 2, Salada Lagoon; 3, Quepiaco Salar; 4, Amarilla Lagoon; 5, Helada Lagoon. **(C)** Principal coordinates analysis of the Jaccard distances based on DNA. **(D)** Principal coordinates analysis of the Jaccard distance between protists lineages detected through DNA and cDNA for three stations. Stars indicate stations with protist communities being significantly different from each other and from the non-labeled stations (for details see text).

**Figure 3:**
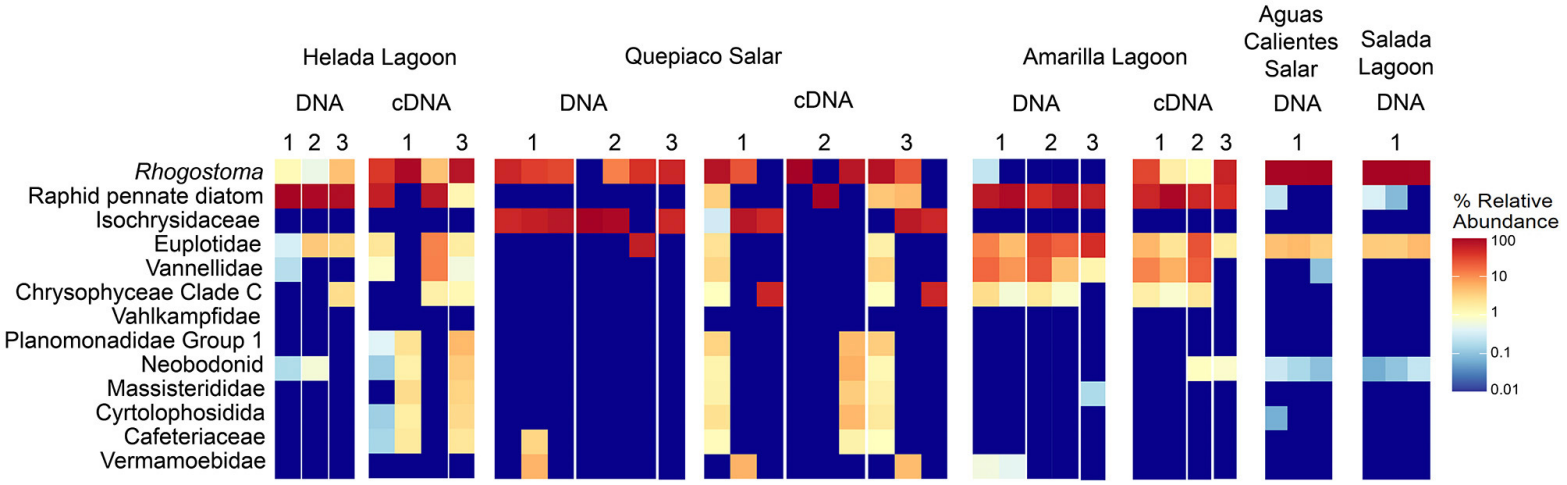
Heatmap of the relative abundance of the top 13 taxa detected in the studied layers from microbial mats based on DNA or cDNA.

The protist communities determined by the DNA metabarcoding were mainly dominated by Rhizaria (15.1–95.6% of the reads), Stramenopiles (0.5–69.4%), Alveolata (5–22.3%), Amoebozoa (0.9–11.3%), and Haptista, which occurred only at Quepiaco Salar (61.5% of the reads) (Figure 2). The dominant taxa detected through cDNA metabarcoding (three study sites) were Stramenopiles, ranging between 19.8% of reads for Quepiaco Lagoon, 34.4% for Helada Lagoon, and up to 59.5% for Amarilla Lagoon. Other important taxa detected by cDNA metabarcoding were Rhizaria (22–51.6% of the reads), Alveolata (6.3–11.9%), and Discoba (0.4–5.5%). Further information on the relative abundance of protist taxa that could be detected across the stations studied by DNA and cDNA is shown in Figure 3.

**Figure 4:**
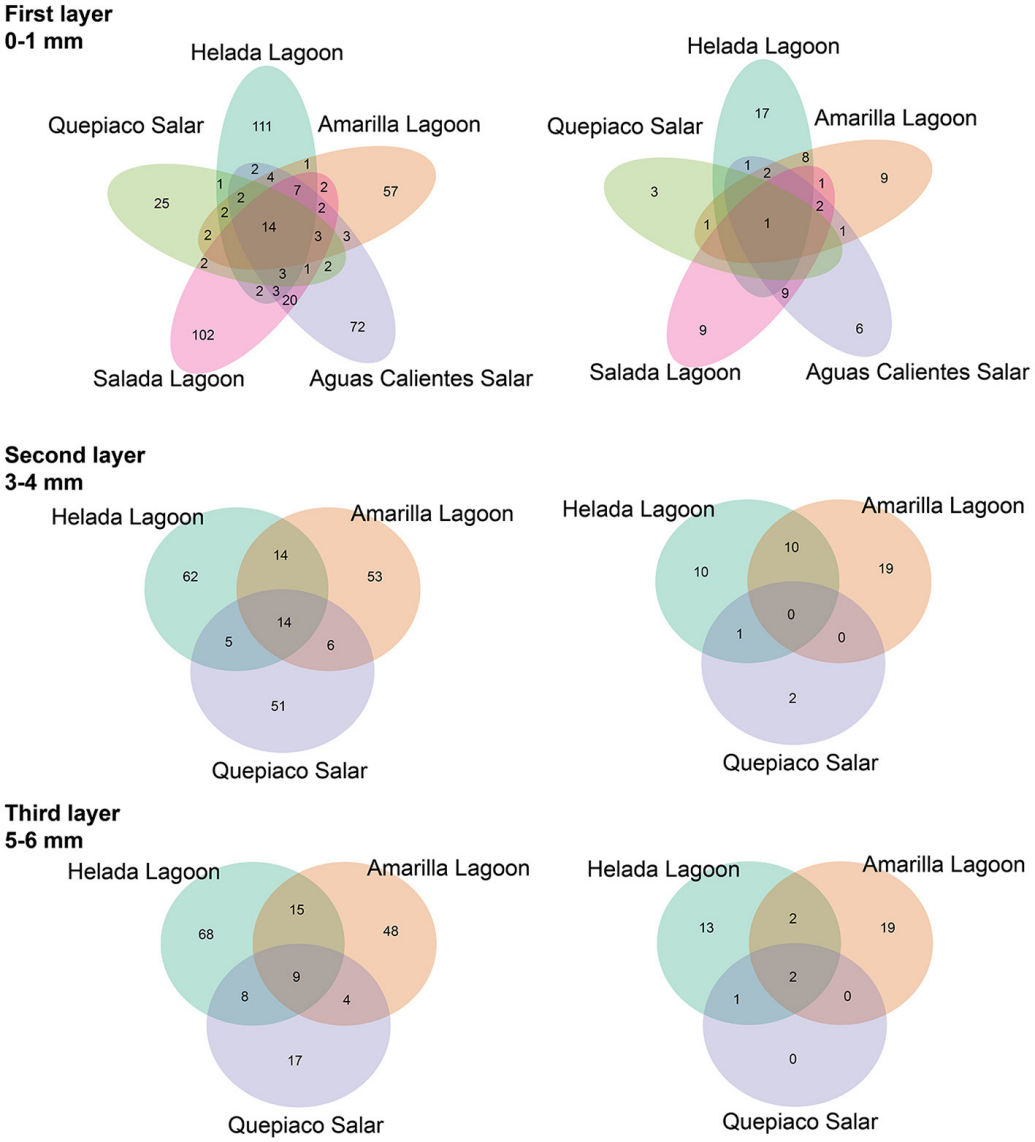
The shared amplicon sequence variants (ASVs) at each layer between stations are detected by DNA metabarcoding. **(Left)** Data without rigid filtering. **(Right)** Data filtered using the mock community-deduced threshold.

The analysis of ASVs of the DNA data sets shared between the different stations revealed a relatively low number either when comparing the different sites or when comparing different layers (Figure 4). The relative number of shared sequences is relatively similar when comparing unfiltered filter data sets or when a rigid (mock) filter is applied.

### Alpha diversity across the five Andean microbial mats

**Figure 5:**
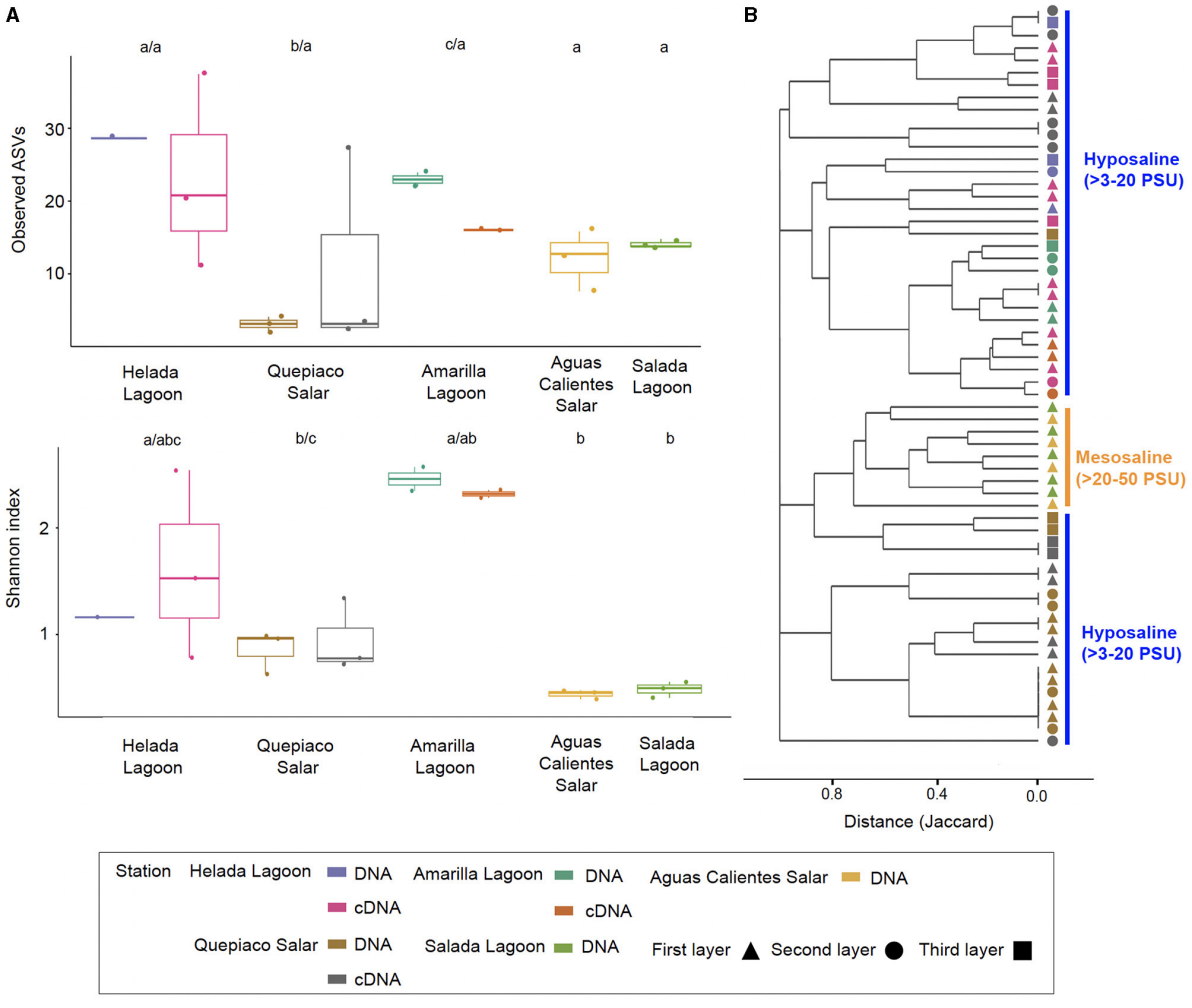
Alpha and beta diversity metrics were measured across the five stations. **(A)** Box and whiskers plots of the observed ASVs and the Shannon index measured in the first layer of microbial mats across stations. Different letters above the boxes mark significant differences, before the slash for DNA, and behind for cDNA data (Kruskal-Wallis, *p* < 0.05). **(B)** Cluster analysis based on the Jaccard distances between protist communities from each layer of microbial mats among stations (*p* < 0.05 for values >0.8). Salinity ranges are indicated on the right side.

The overall mean number of ASVs was 10.8 with a standard deviation (SD) of 7.1, with the highest mean ASVs obtained from Amarilla Lagoon (mean = 20.2, SD = 1.1) and the least from Quepiaco Salar (mean = 2.5, SD = 1.1) (Figure 5A). The overall mean Shannon diversity obtained was 1.2 with a standard deviation of 1.4, with the highest value for Amarilla Lagoon (mean = 3.3, SD = 0.2) and the lowest for Aguas Calientes Salar (mean = 0.5, SD = 0.06). The overall phylogenetic diversity mean value was 15.7 with a standard deviation of 5.3, with the highest mean value found for Amarilla Lagoon (mean = 18.6, SD = 6.05) and the lowest for Quepiaco Salar (mean = 10.7, SD = 0.6). We tested the dissimilarity between the groups of protists detected across the study sites, considering the observed ASV numbers and Shannon diversity calculated from the DNA as well as from cDNA data sets. The amount of observed ASVs was significantly different between Amarilla Lagoon and all other study sites, and that of Quepiaco Salar was also significantly different from all the other study sites (Figure 5A). When we compared the study sites regarding the Shannon index, significant differences were found between Quepiaco Salar and Helada Lagoon on the one side and the other three study sites on the other side (Figure 5A). Regarding the results of the cDNA metabarcoding data for the three study sites investigated, there were no significant differences regarding the number of observed ASVs, while the data from Quepiaco Salar differed from the other two study sites regarding the Shannon index. The cDNA values for one station clustered very similar to the respective DNA values when compared using the principal coordinate analysis (Figure 2D).

### The structure of protist communities detected through DNA and cDNA

The dissimilarity measured between the studied layers across stations is presented in a cladogram based on the Jaccard distance, which indicates four main groups (Figure 5). Two groups were composed of samples from Quepiaco Salar (DNA and cDNA), Aguas Calientes Salar, and Salada Lagoon (DNA), and two groups were composed of Helada Lagoon, Amarilla Lagoon, and Quepiaco Salar. This last group includes samples obtained from both DNA and cDNA from Helada Lagoon and Amarilla Lagoon and samples obtained from cDNA at Quepiaco Salar (Figure 5). The principal coordinate analysis (PCoA; Figure 2C) explains 53.2% (PCo1 = 30.2%, PCo2 = 23%) of the dissimilarity between the five study sites (DNA). On the other hand, the protist communities analyzed by the DNA and cDNA for three study sites (Figure 2D) showed a pattern of dissimilarity explained by 48% (PCo1 = 31.5%, PCo2 = 16.5%). However, the differences between the communities detected through DNA and cDNA within samples from one station were not significant in all three investigated cases.

### Association of the abiotic factors and the protist communities

**Figure 6:**
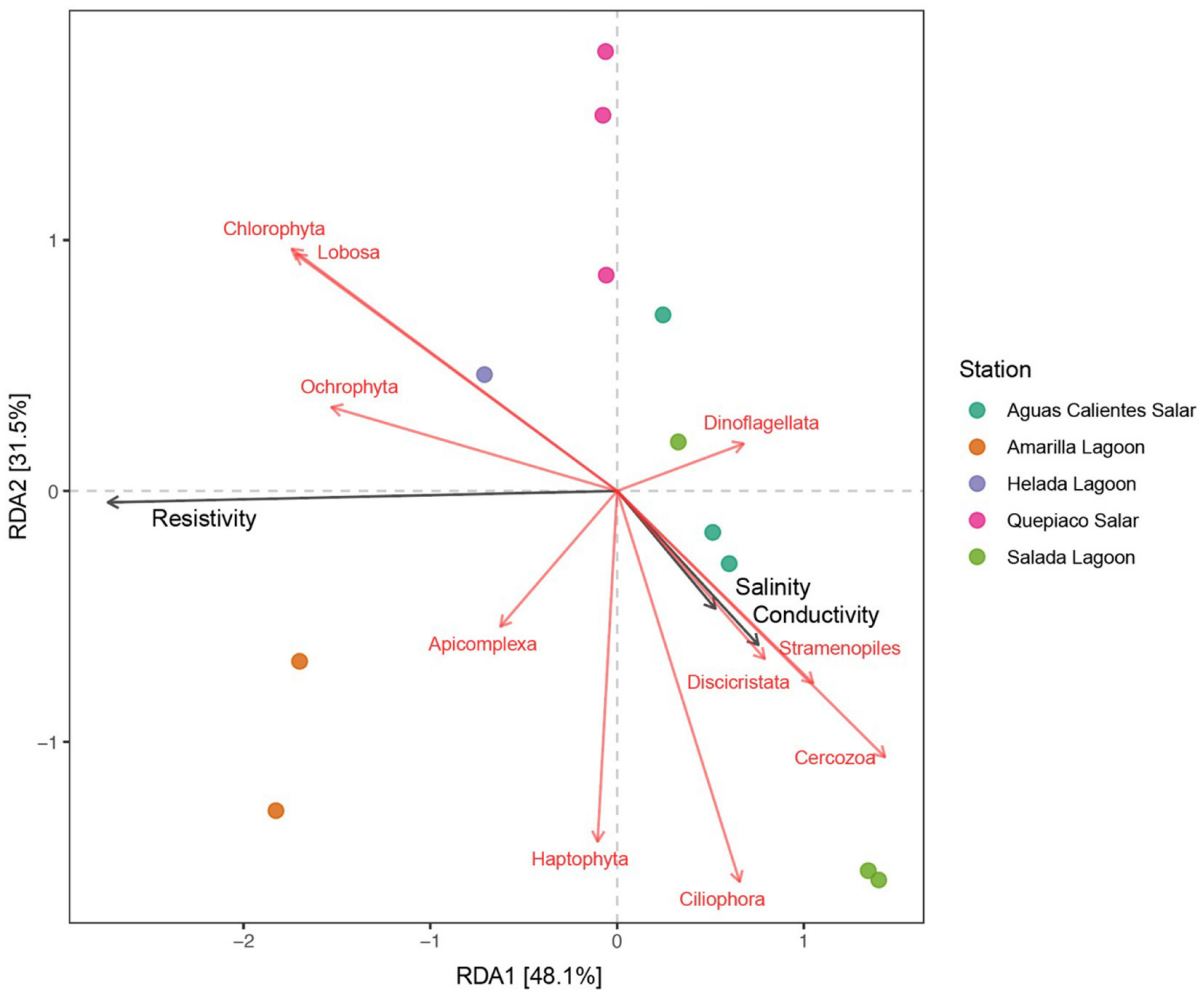
Canonical redundancy analysis (RDA) of the protist genotypes detected in the upper layer of the studied microbial mats (identity to the database > 97%). Black arrows represent the environmental variables that have a significant effect on the studied genotypes and the red arrows represent the genotypes associated with the environmental variables.

The results of the redundancy analysis (RDA), including the protist lineages of the DNA metabarcoding data sets in the first layer across the five stations, revealed significant correlations (Figure 6). On the other hand, the analysis of cDNA data detected no significant association between the protist taxa and environmental factors. The interpretation rate of the RDA for DNA metabarcoding was equal to 79.6% (RDA1 = 48.1%, RDA2 = 31.5%). The resistivity (*R*^2^ = 0.8818, Pr = 0.001), the conductivity (*R*^2^ = 0.7259, Pr = 0.003), and the salinity (*R*^2^ = 0.7231, Pr = 0.003) of the water bodies were selected as significant factors (*p* < 0.01) associated with the protist composition of the studied microbial mats. Furthermore, the protist taxa associated with these environmental parameters were chlorophytes, Lobosa (Amoebozoa), Cercozoa (Rhizaria), Ciliophora, Apicomplexa and Dinoflagellata (Alveolata), Ochrophyta, Haptophyta, Discicristata, and one unclassified division of Stramenopiles.

## Discussion

In this study, we delve into the ecology of protists in the microbial mats of five Andean Lagoons with different salinities, characterizing the structure of their communities through the amplification of the V9 region (18S SSU). The primary goal was to assess, at the millimeter scale (Figure 1), the potentially resting stages as well as the active protist communities detected through the metabarcoding of DNA and cDNA in five stations. Partly, this could not be resolved, as the amplification of the target gene was successful mainly from the upper layers and from the DNA and cDNA templates only in three stations (Figure 3). Future studies aiming to study the stratification and activity of protist groups in Andean microbial mats should consider the use of both hypervariable regions (V4 and V9), as recommended for comprehensive diversity studies using environmental sequence variants (Choi and Park, 2020). Nevertheless, with our study, we could determine the ASVs shared across all stations at each studied layer (Figure 4). The restricted overlap between the different microbial mats indicates the specificity of the different aquatic habitats. The reasons for this specificity could not be fully explained in the course of the present study; however, salinity might be an important driver (see below). Overall, phototrophs and amoebae are frequent in the uppermost layers, and ciliates are common throughout all depths (DNA metabarcoding). The active protist groups (cDNA) were mainly diatoms shared between stations at the first (0–1 mm) and second (3–4 mm) layers, while *Euplotes* was the only genotype detected as active and shared by the deepest layer (5–6 mm). The functional diversity of microbes can vary across the layers of microbial mats due to changes in water and light availability, particularly for oxygen producers such as the cyanobacteria and diatoms studied in the saline lake of the Atacama Desert, the Salar de Llamará (Demergasso et al., 2003). Such variations could be responsible for the observed differences in the vertical structure of protist communities. As initially hypothesized, the studied ecosystems appeared to be highly underexplored regarding protists lineages. ASVs classified as eukaryotes accounted for < 1% of the total sequences (at 97% identity in databases). Moreover, using a lower identity threshold (80% of identity to the databases), only up to 7.7% of the total sequences could be assigned to protist lineages. This supports the hypothesis of a high novelty in such remote ecosystems. This is in agreement with Burki et al. (2021), who suggest that such remote and underexplored environments could constitute an untapped reservoir of potential new biodiversity. Our RDA analysis revealed that factors such as salinity, conductivity, and resistivity were correlated with the detected benthic communities, which included cercozoans, ciliates, lobose amoebae, diatoms, and kinetoplastids (Figure 6). Among these, salinity is a factor often related to desiccation, shaping the community structures of microbial communities under water fluctuations (Rothrock and Garcia-Pichel, 2005; Lozupone and Knight, 2007). Other abiotic factors, such as conductivity, solar radiation, and temperature, have been associated with the community composition of microbes in hypersaline lagoons, mainly linking salinity to microbial abundance (Bryanskaya et al., 2016). Based on our results, we underscore the potential of high-throughput sequencing approaches to explore the structure of protists communities thriving in delicate and complex habitats and the limitations of the study of protists living at elevated salinity.

One essential component of biodiversity surveys using metabarcoding has been the use of an artificial sample (a mock community), aiming to identify spurious sequences (Schloss et al., 2011). This has already been implemented in the study of aquatic ecosystems throughout the Atacama and the search for patterns at the community level without blurring the diversity patterns (Rybarski et al., 2023). In our study, we could assess the taxonomy of the artificial samples in three sequencing libraries for the studied microbial mats and could define a frequency threshold deduced from the frequency of reads with low abundances. We used the frequency of these reads to filter ASVs of small abundance in our environmental samples (frequency < 0.032–0.047%), affecting mainly the ASV numbers (Table 2). Approximately 9.6–18.9% of the total ASVs and between 82 and 95.2% of sequences passed this filter and were included in the diversity assessment in the downstream analyses. The correct identification of the eight strains in the mock communities additionally supported the classification method for the study of our environmental samples. With these results, we confirm the use of mock community-deduced thresholds as the backbone of studies on protist communities in different environments through metabarcoding (e.g., Fiore-Donno et al., 2019; Dünn and Arndt, 2023; Sachs et al., 2023).

### Alpha diversity and taxonomy of protists across five microbial mats

Studies on the protist diversity using metabarcoding in the Atacama Desert and the Andean region are scarce. Our results are comparable to one study on the protists diversity (hypervariable region V4) in microbial mats from the hypersaline lagoons in Llamará Salar (Saghaï et al., 2017). We can also compare our work to one study of heterotrophic flagellate communities (hypervariable region V9), detected in the waters of diverse salars across the Atacama (Rybarski et al., 2023). Those authors found a diversity of protists, reaching a minimal amount of six and up to 214 OTUs per study site. In our study, we registered lower numbers ranging between 2 and 20 ASVs per replicate. The amount of shared and unique biosignatures per station was similar (our study: 1–11 ASVs; Rybarski et al., 2023: 1–44 OTUs). Our samples reached a mean number of ASVs that could be confirmed by cDNA studies at the referred stations, reaching similar values at each station (e.g., Figure 5). Furthermore, our analysis of the shared and unique communities, including raw sequences, showed similar numbers of observed shared and unique genotypes as obtained by Rybarski et al. (2023). The registered diversity in Llamará Salar microbial mats (Saghaï et al., 2017) is low at stations of higher salinity (Simpson diversity index 0.93). In accordance with this, our results showed that the protist communities detected through DNA and cDNA metabarcoding include a low diversity at stations with elevated salinity (Figure 5).

At the millimeter scale, we detected abundant raphid diatoms, mainly in the upper layers of the microbial mats in the lagoons of Helada and Amarilla. In the active community assessment (cDNA), they appear abundant in the upper layers (Supplementary Figure 2). Phototrophs are the main builders of microbial mats, contributing to their formation through the excretion of exopolymeric substances (EPSs) and by facilitating carbonate accumulation in organo-mineralization processes. In our dataset, we identified *Amphora, Navicula*, and *Nitszchia* as primary producers, which agrees with the findings from Saghaï et al. (2017). Their study documented a high occurrence of diatoms in the upper layers of microbial mats in another nearby Atacama saline lake, studied at the centimeter scale, as well as in other Andean microbial mats (Farías et al., 2014; Albarracín et al., 2015; Vignale et al., 2022). Among stramenopiles, we also detected ASVs closely related to genotypes of marine diatoms (e.g., *Skeletonema, Thalassiosira, Phaeodactylum*), freshwater diatoms (e.g., *Cymbella, Pseudogomphonema*), chrysomonads (e.g., *Paraphysomonas, Ochromonas*) and diverse unclassified raphid pennate diatoms. Saghaï et al. (2017) also found stramenopiles accompanied by choanoflagellates, bicosoecids, and ciliates, with small numbers of OTUs dominating the abundance of the protist communities. In deep layers, they detected amoebae, with Conosa reaching almost 10% of the relative abundance of protists. Interestingly, in our study in the deepest layer of Quepiaco Salar, amoebae were also detected as being active and abundant (Supplementary Figure 2, Quepiaco Salar cDNA). We assessed the shared communities of protists throughout the first, second, and third layers across the stations (Figure 4). In the first layer, a common amoeba in the DNA and cDNA sets is the thecate amoeba *Rhogostoma*. Other taxa detected through cDNA in the first layer (Helada Lagoon, Amarilla Lagoon, and Quepiaco Salar) include *Amphora, Euplotes, Vannella*, and raphid pennate diatoms. No shared protists were found in the second layer; however, *Euplotes, Vannella*, and *Navicula* genotypes occurred in the unfiltered data set among the shared taxa (Helada and Amarilla Lagoons, Quepiaco Salar). At the deepest layer, a genotype of the ciliate genus *Euplotes* appeared to be shared in both DNA and cDNA data sets (Supplementary Figure 2), being abundant and represented by highly similar ASVs (one nucleotide difference, Supplementary Table 3). One ASV was 100% identical to a strain isolated from the Amarilla Lagoon (HFCC988, *Euplotes* sp.). This speciose genotype, or at least a very similar one, was also reported from Llamará Salar by Saghaï et al. (2017).

A typical protistan genotype we found at the different stations belonged to the genus *Rhogostoma*, a common protist genotype that we identified through DNA and cDNA analysis and occurred mainly in the uppermost layers of the microbial mats. Species of this genus are common inhabitants of aquatic and terrestrial ecosystems (Öztoprak et al., 2020). Although we cannot indicate the presence of this genotype as a species, we can trace our result to one cercozoan genus registered earlier as an arboretum inhabitant (Howe et al., 2011) and recently to a species member of the phyllosphere of endemic cacti in the Atacama (Acosta et al., 2023). This thecofilose amoeba, *Rhogostoma*, likely predates phototrophs as the diatoms (e.g., *Amphora*) are detected in parallel. Other remarkable protist lineages are those taxa detected in Quepiaco Salar, represented by *Cafeteria, Isochrysis*, and *Hartmannella*, of which the first two include marine strains (Bendif et al., 2013; Schoenle et al., 2020). The occurrence of the genus *Cafeteria* has already been reported from the Atacama Desert (Schoenle et al., 2022).

### Factors associated with the benthic protists

The clustering patterns of the detected protist communities are significant based on the distance metrics shown in our PCoA analyses (Figure 2). The active DNA pool (cDNA) differed between the stations but corresponded to the total DNA pool of the respective station. However, we found, at least in part, indications for layer specificity in the activity of protists (Figure 5). Phototrophs were mainly detected in the upper layers, which supports the existence of a vertical gradient reflected in the studied distribution patterns.

Furthermore, we found that salinity plays an important role in the community structure of protists registered by metabarcoding for the different microbial mats. Differences in the salinity of the water bodies originated from different catchment areas (Ritter et al., 2018), explaining in part the uniqueness of environmental conditions in each salar/lagoon and the differences in community structure (Figure 6). The salinity is specifically tolerated by protist species from various phylogenetic lineages isolated from hypersaline lagoons and salars of the Atacama (Schiwitza et al., 2018, 2019, 2021; Carduck et al., 2021; Rybarski et al., 2021; Schoenle et al., 2022).

Another important factor that might have governed the differences in the protist community structure observed in our studies could be the differences in the community structure of prokaryotes, which are known to have a significant effect on protist communities (e.g., Glücksman et al., 2010). It has been shown that the community structure of prokaryotes can differ between the different aquatic habitats across the Atacama and the Andes, as shown by Demergasso et al. (2003), Dorador et al. (2008), and in later studies by Rasuk et al. (2014) and Farias (2016). As salinity levels may vary due to changes in water availability, the composition of active and resting taxa within these ecosystems can change significantly, altering the biodiversity of these natural ecosystems. Future studies, including the metabarcoding of 16S and 18S rRNA phylogenetic markers, could help to elucidate overall changes in the community structures of protists detected co-occurring with the prokaryotes typically found in microbial mats at different salinities characterizing the Andean range.

## Data availability statement

The datasets presented in this study can be found in online repositories. The names of the repository/repositories and accession number(s) can be found below: NCBI—PRJNA1062042.

## Author contributions

EA: Data curation, Formal analysis, Investigation, Visualization, Writing – original draft. FN: Data curation, Conceptualization, Methodology, Supervision, Writing – review & editing. CD: Writing – review & editing, Resources. HA: Writing – review & editing, Conceptualization, Data curation, Methodology, Project administration, Supervision.
