# Supplementary material for: Protist communities of microbial mats from the extreme environments of five saline Andean lagoons at high altitudes in the Atacama Desert
Source: Front Microbiol. 2024 Mar 20;15:1356977. doi: 10.3389/fmicb.2024.1356977 (PMC10987879; doi:10.3389/fmicb.2024.1356977)
Supplement: Supplementary file 1 [file Data_Sheet_1.pdf]

frontiers

Supplementary Material

1 Supplementary Figures and Tables

1.1 Supplementary Tables

Supplementary Table 1. List of the strains from the Heteroflagellate Collection Cologne (HFCC) included in the mock communities sequenced during this work.

Species	HFCC	Expedition	Accession No.
**ALVEOLATA**			
Protocruzia sp.	766	M139	MT355146
Aristerostoma sp.	744	M139	MT081566
**ANCYROMONADIDA**			
Fabomonas tropica	175	SO237	MT355148
**RHIZARIA**			
Massisteria sp.	176	SO237	MT355122
**DISCOBA**			
Rhynchomonadidae sp.	171	SO237	MT355133
**STRAMENOPILA**			
Cafeteria burkhardae	203	SO237	MN315604
Bicosoecida	768	M139	MT355117
**OPISTHOKONTA**			
Ministeria vibrans	1787	SO237	MT355150

Supplementary Table 2. Identification of abundant ASVs detected by metabarcoding and their identification according to the Protist Ribosomal Reference PR^2 and the best hit in BLAST (NCBI).

ASV	Sequence	PR^2	Best hit BLAST (NCBI)	Accesion Number
0aeb77319dc51b7c5679cee98aac6cbb	CACACCGCCCGTCGCTCCTACCAATTTCGAGTGGCTCGGTGAACCTCTTTGGACTGTCGAGCAATCGCGAAATTAGAGTGAACCTG GTCACTTAGAGGAAGGAGAAGTCGTAACAAGGTTTCCGTAGGTGAACCTGCG	*Euplotes*	*Euplotes* sp. IsolateB528	KX516712
86ed9995164224c825234657c61e1115	CACACCGCCCGTCGCTCCTACCAATTTCGAGTGGCTCGGTGAACCTCTTTGGACTGTCGAGCAATCGCGAAATTAGAGTGAACCTG GTCACTTAGAGGAAGGAGAAGTCGTAACAAGGTTTCCGTAGGTGAACCTGCA	*Euplotes*	Uncultured ciliate clone ZOTU20	MT095067
26853f2f68bfbf6dd9a98111bac970ba	ACCGCCCGTCGTTGTTTCCGATGATGGTGCAATACAGGTGATCGGACTGACGAGCCCCTGGCTTGTCGGAAAGTTCACCGATATTT CTTCAATAGAGGAAGTAAAAGTCGTAACAAGGTAGCTGTAGGTGAACCT	*Neobodo designis*	Uncultured eukaryote clone L10.2	AY753968
2c4aa5564987eeeb63d9a675464358ee	ACCGCCCGTCGTTGTTTCCGATGATGGTGCAATACAGGTGATCGGATAGATGGGTGTTTCACTTGTCTAGAAGTTCACCGATATTTC TTCAATAGAGGAAGCAAAAGTCGTAACAAGGTAGCTGTAGGTGAACCT	*Neobodon id*	Cryptaulaxoi des-like sp. TCS-2003	AY425021
7cd0432cb279182befc56bdc71b6bab6	ACCGCCCGTCGTTGTTTCCGATGATGGTGCAATACAGGTGATCGGACTGACGAGCCTCTGGCTTGTCGGAAAGTTCACCGATATTT CTTCAATAGAGGAAGTAAAAGTCGTAACAAGGTAGCTGTAGGTGAACCT	*Neobodo designis*	Uncultured eukaryote clone L10.2	AY753968
af31100e08189a6c6c0bb559c9610286	ACCGCCCGTCGTTGTTTCCGATGATGGTGCAATACAGGTGATCGGACAGACGAACCTCTGGTTTGTCTGAAAGTTCACCGATATTTC TTCAATAGAGGAAGTAAAAGTCGTAACAAGGTAGCTGTAGGTGAACCT	*Neobodo designis*	Neobodo designis isolate Dune	AY753622
f62c19f04b9561776eb24fe24e3a94b5	CGCCCGTCGTTGTTTCCGATGATGGTGCAATGCAGGTGATCGGACAAGCAGAGGCTCTGTCTTTGCTTGAAAGTTCACCGACATTTC TTCAATAGAGGAAGCAAAAGTCGTAACAAGGTAGCTGTAGGTGAAC	*Rhyncho monas nasuta*	Rhynchomo nadidae sp. HFCC173	MT355135
f1b89a1002956d332262728c89fa7e85	GTCGCTACTACCGATTGAATGGCTTAGTGAGCTCTCTGGGCTGTTGCGTGTTGGGGGCAACTCCGTCACGCAAACGGGAAGGAGAT CAAACTTGATCATTTAGAGGAAGTAAAAGTCGTAACAAGGTTTCC	*Rhogosto ma-* lineage sp	*Rhogostom a* sp. isolate HFCC860	OR775727
e4876cbac4554cc47a78d0987bb83174	GTCGCTACTACCGATTGAATGGCTTAGTGAGCTCTCTGGACTGTTGCGTGTTGGGGGCAACTCCGTCACGCAAACGGGAAGGAGAT CAAACTTGATCATTTAGAGGAAGTAAAAGTCGTAACAAGGTTTCC	*Rhogosto ma-* lineage sp	*Rhogostom a* sp. isolate HFCC860	OR775727
db192667fc0011815785aa6757854e82	GTCGCTACTACCGATTGAATGGCTTAGTGAGCTCTCTGGACTGTTGCGTGTTGGGGGCAACTCCGTCACGCAAACGGGAAGGAGAT CAAACTTGATCATTTAGAGGAGGTAAAAGTCGTAACAAGGTTTCC	*Rhogosto ma-* lineage sp	*Rhogostom a* sp. isolate HFCC860	OR775727
26ea63322615c90fd06dc5395c434ef9	GTCGCTACTACCGATTGAATGGCTTAGTGAGCTCTCTGGACTGTTGCGTGTTGGGGGCAACTCCGTCACGCAAACGGGGAGGAGAT CAAACTTGATCATTTAGAGGAAGTAAAAGTCGTAACAAGGTTTCC	*Rhogosto ma-* lineage sp	*Rhogostom a* sp. isolate HFCC860	OR775727
5fbe65945d8bc635112c40fe0889ada8	GTCGCTACTACCGATTGAATGGCTTAGTGAGCTCTCTGGACTGTTGCGTGTTGGGGGCAACTCCGTCACGCAAACGGGAAGGAGAT CAAGCTTGATCATTTAGAGGAAGTAAAAGTCGTAACAAGGTTTCC	*Rhogosto ma-* lineage sp	*Rhogostom a* sp. isolate HFCC860	OR775727
4ae9c1b363f7004798d51a63ff8842a5	GTCGCTCCTACCGATTGAATGATCCGGTGAGGCCCCCGGACTGCGGCTCCGTCGCTGGTTCTCCAGCTTTGGGGTTGCGGGAAGC TGTCCGAACCTTATCATTTAGAGGAAGGAGAAGTCGTAACAAGGTTTCC	*Isochrysis galbana*	*Isochrysis galbana* strain CCAP 927/1	KU900225
e26dbcae054f24c3de164299928a5b82	GTCGCTGCTACCGATTGGATGGTCCGGTGAAATCCTCGGATAGTGAAGATCACTTTAACCTTCGGGTTGAAGCTCATTGCTAGAAGT TGATTAAACCTTATCATCTAGAGGAAGCAAAAGTCGTAACAAGGTTTCC	*Vannella contorta*	*Platyamoeb a contorta* isolate W51C#5	DQ229954

4537164385a9f4f04bdc1d4e68247f44	GTCGCACCTACCGATTGAATGGTTCGGTGAAACTTTCGGACCGTGGACAGGTCGCCTTCGGGCGACTTGACCGTGGGAAGTTATTTAAACCTCATCATTTAGAGGAAGGTGAAGTCGTAACAAGGTTTCC	Chrysophyceae Clade C	*Pedospumella* sp. JBM19	KX442747
36746617900e43b4ec1db7601939cb8a	GCCCGTCGCTCGTACCGACTGAATAGAGGTATGAATTTGATGGACCAGCGTTGCATCAAGCAATGCGAGAAAATCAACTGAATTTCTCTATTTGTAGGAACGAGAAGTCGTAACAAGGTCTTCGTAGGTGAA	Vahlkampfidae	*Tetramitus dokdoensis 18S*	KY463322
53eb12cdee4e38ed83b2a6e8bc14f439	GTCGCTCCTACCGATTGGATGGTCCGGTGAAATCTTCGGACTGCAGCGGCGCAGCTGGTTTTCCGGCAGTGCCGCAGCGGGAAGTTGCTTAAACCTTATCATCTAGAGGAAGGAGAAGTCGTAACAAGGTTTCC	Planomonadidae	*Fabomonas tropica*	MW872733

1.2 Supplementary Figures

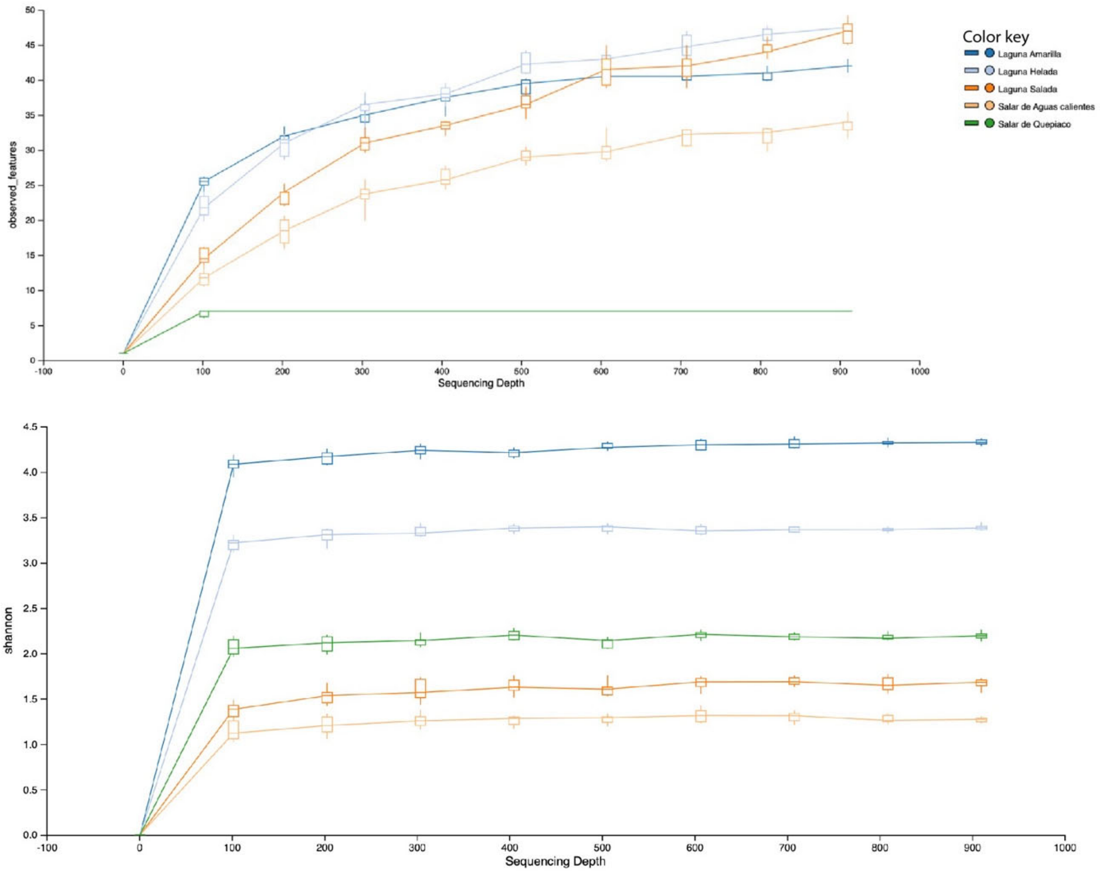

Supplementary Figure 1. Rarefaction curves obtained from the sequencing in all stations included in this study showing the number of ASVs as function of the number of sequenced reads.

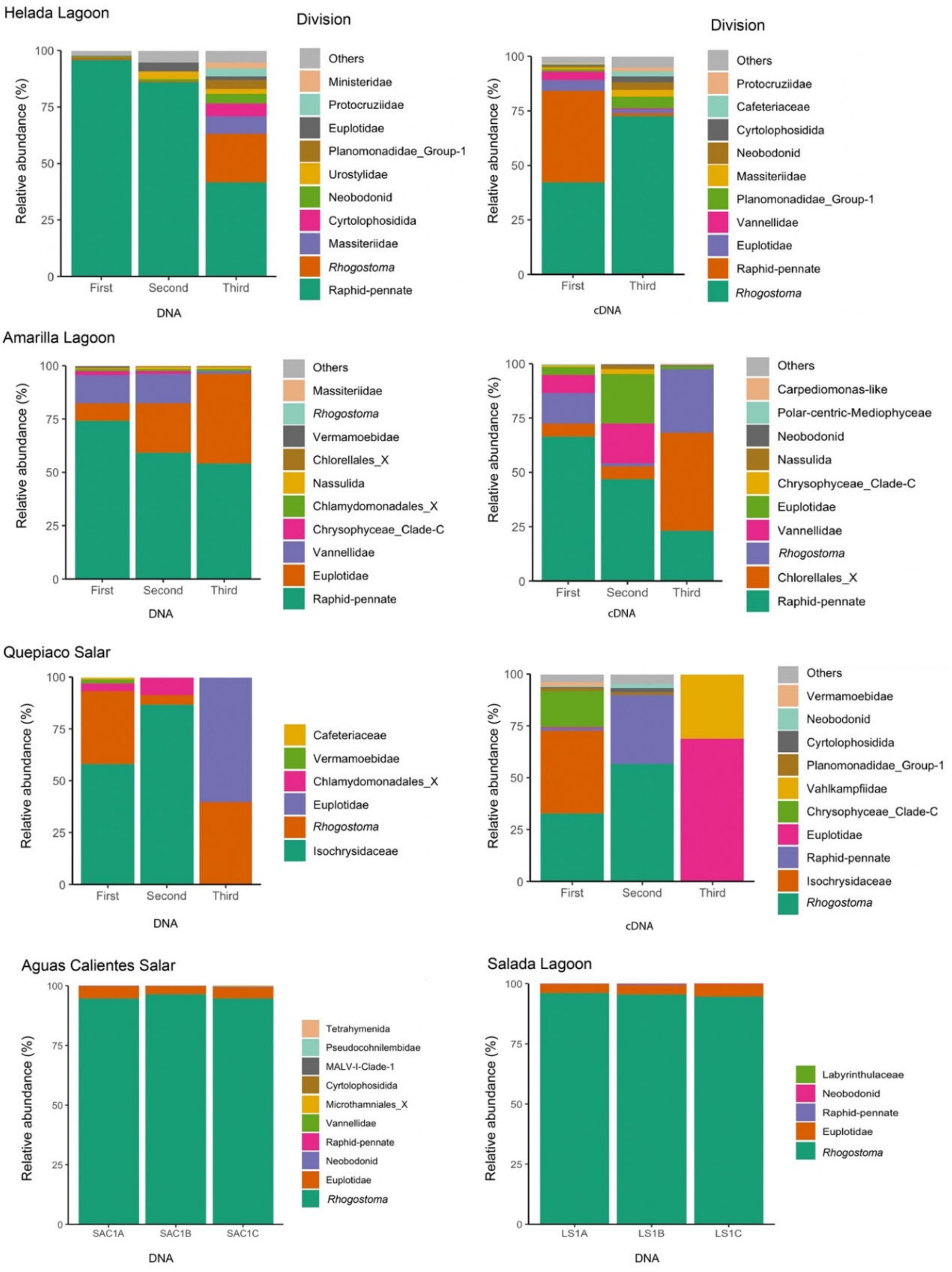
Helada Lagoon
Division
Relative abundance (%)
100
75
50
25
0
First
Second
Third
DNA
Others
Ministeriidae
Protocruziidae
Euplotidae
Planomonadidae_Group-1
Urostylidae
Neobodonid
Cyrtolophosidida
Massiteriidae
Rhogostoma
Raphid-pennate
Division
Relative abundance (%)
First
Third
cDNA
Others
Protocruziidae
Cafeteriaceae
Cyrtolophosidida
Neobodonid
Massiteriidae
Planomonadidae_Group-1
Vannellidae
Euplotidae
Raphid-pennate
Rhogostoma
Amarilla Lagoon
Relative abundance (%)
First
Second
Third
DNA
Others
Massiteriidae
Rhogostoma
Vermamoebidae
Chlorellales_X
Nassulida
Chlamydomonadales_X
Chrysophyceae_Clade-C
Vannellidae
Euplotidae
Raphid-pennate
Relative abundance (%)
First
Second
Third
cDNA
Others
Carpediomonas-like
Polar-centric-Mediophyceae
Neobodonid
Nassulida
Chrysophyceae_Clade-C
Euplotidae
Vannellidae
Rhogostoma
Chlorellales_X
Raphid-pennate
Quepiaco Salar
Relative abundance (%)
First
Second
Third
DNA
Cafeteriaceae
Vermamoebidae
Chlamydomonadales_X
Euplotidae
Rhogostoma
Isochrysidaceae
Relative abundance (%)
First
Second
Third
cDNA
Others
Vermamoebidae
Neobodonid
Cyrtolophosidida
Planomonadidae_Group-1
Vahlkampfiidae
Chrysophyceae_Clade-C
Euplotidae
Raphid-pennate
Isochrysidaceae
Rhogostoma
Aguas Calientes Salar
Relative abundance (%)
SAC1A
SAC1B
SAC1C
DNA
Tetrahymenida
Pseudocohnilembidae
MALV-I-Clade-1
Cyrtolophosidida
Microthamniales_X
Vannellidae
Raphid-pennate
Neobodonid
Euplotidae
Rhogostoma
Salada Lagoon
Relative abundance (%)
LS1A
LS1B
LS1C
DNA
Labyrinthulaceae
Neobodonid
Raphid-pennate
Euplotidae
Rhogostoma

Supplementary Figure 2. Relative abundance of the protist groups detected across layers of the studied microbial mats through DNA and cDNA. Aguas Calientes and Salada Lagoon include the three replicates detected in the first layer of the microbial mats through DNA.
